# Association between periodontitis and dental caries: a systematic review and meta-analysis

**DOI:** 10.1007/s00784-024-05687-2

**Published:** 2024-05-10

**Authors:** Yixin Li, Yonggang Xiang, Haixia Ren, Chao Zhang, Ziqiu Hu, Weidong Leng, Lingyun Xia

**Affiliations:** 1 Department of Stomatology, Taihe Hospital, Hubei University of Medicine, Shiyan, 442000 Hubei China; 2 Department of Ophthalmology, Taihe Hospital, Hubei University of Medicine, Shiyan, 442000 Hubei China; 3 Center for Evidence-Based Medicine and Clinical Research, Taihe Hospital, Hubei University of Medicine, Shiyan, 442000 Hubei China

**Keywords:** Periodontitis, Dental caries, Oral bacteria, Antagonistic, Systematic review

## Abstract

**Objectives:**

Recent evidence suggested a link between periodontitis (PD) and dental caries, but the trends and nature of this association remained unclear. The overall aim of this study was to critically assess the correlation of two disorders.

**Methods:**

A comprehensive search was conducted within the PUBMED and EMBASE databases including grey literatures up to July 5th, 2023. The Newcastle–Ottawa scale was used to qualitatively evaluate the risk of bias.

**Results:**

Overall, 18 studies were included. In terms of caries risk in PD patients, the prevalence of caries was increased by PD (OR = 1.57, 95%CI:1.20–2.07), both in crown (OR = 1.03, 95%CI:1.01–1.05) and root caries (OR = 2.10, 95%CI:1.03–4.29). Odds of caries were also raised by PD severity (OR _moderate_ = 1.38, 95%CI:1.15–1.66; OR _severe_ = 2.14, 95%CI:1.74–2.64). Besides, patients with PD exhibited a higher mean number of decayed, missing and filled teeth (DMFT) and decayed and filled root teeth (DFR) [weighted mean difference (WMD)_DMFT_ = 0.87, 95%CI: -0.03–1.76; WMD_DFR_ = 1.13, 95%CI: 0.48–1.78]. Likewise, patients with caries had an elevated risk of PD (OR = 1.79, 95%CI:1.36–2.35). However, Streptococcus mutans, one of the main pathogens of caries, was negatively correlated with several main pathogens of periodontitis.

**Conclusions:**

This study indicated a positive correlation between dental caries and periodontitis clinically, while the two disease-associated pathogens were antagonistic.

**Clinical relevance:**

Further research, including clinical cohort studies and mechanisms of pathogens interaction is needed on this link for better prevention and treatment of PD and caries. In addition, innovative prevention strategies need to be developed and incorporated in dental practices to prevent these two highly prevalent oral diseases.

**Supplementary Information:**

The online version contains supplementary material available at 10.1007/s00784-024-05687-2.

## Introduction

Periodontitis (PD) is a chronic multifactorial inflammatory disease caused by dysbiosis, resulting in progressive destruction of the dental surrounding tissues and tooth loss [1]. Its severe form was considered the 6th most prevalent disease of humankind in 2010, which affected 743 million people aged 15 and over worldwide [2, 3]. Dental plaque is an initiating factor that first causes gingivitis if accumulated, resulting in the loss of collagen locally [4]. Destructive periodontitis will occur gradually if the inflammation is not well controlled, which has been associated with dysbiosis where the diversity, richness and relative proportions of species in the subgingival microbiota are altered [5]. While the periodontal diseases are unquestionably initiated by bacteria, it is the individual’s host inflammatory response and other risk factors that ultimately determine the clinical presentation and outcome of the many and varied forms of periodontal disease [6]. The epithelium lining periodontal pockets becomes ulcerated, providing a direct entry point for periodontal bacteria into the systemic circulation. Alternatively, the inflammatory response to periodontal bacteria or their by-products may have indirect systemic effects linking periodontitis to a number of chronic systemic diseases [7], such as diabetes [8], rheumatoid arthritis [9] and Alzheimer's disease [10].

Dental caries, otherwise known as tooth decay, is one of the most prevalent chronic diseases among people worldwide [11]. According to the World Health Organization (WHO) report in 2022, untreated carious lesions (both deciduous and permanent teeth), severe periodontal disease, edentulism and cancer of the lip and oral cavity were the leading causes of oral disease burden [12]. It is caused by cariogenic microorganisms in the dental plaque biofilm, which ferment dietary carbohydrates to produce acid, leading to mineral loss from tooth hard tissues and subsequently the destruction of tooth structures [13]. The interplay between microorganisms, diet and host susceptibility determines whether dental caries will occur [11]. Dental caries in enamel is typically first seen as white spot lesions. The cavity site provides an ecological niche in which plaque organisms gradually adapt to a reduced pH [14], followed by bacteria penetrating further into the tissue at an earlier stage of lesion development [15] and gradually progressing to pulpitis, periapical inflammation, and even tooth loss if not controlled [16].

Most of the time, a homeostatic balance exists between the host and microbial communities. Distinct microenvironments contain unique microbial communities, which are regulated through sophisticated signal systems and by host and environment [5]. The dynamic and polymicrobial oral microbiome is a direct precursor of dental caries and periodontitis, two of the most prevalent microbially induced disorders worldwide. As a community develops, microbial metabolism and by-products of the host immune response can cause changes to the local environment that facilitate the outgrowth or over-representation of microorganisms associated with a dysbiotic state [17]. Mutans streptococci (especially Streptococcus mutans) and Lactobacillus have long been recognized as pathogens that are associated with caries [5], where previous studies supported a significant correlation between their concentration in saliva and proportion in plaque [13]. While the flora imbalance of Porphyromonas gingivalis, Fusobacterium nucleatum, Actinobacillus actinomycetemcomitans and others are tied to the progression of periodontitis [18–20]. Due to the distinction of pathogenic bacteria and clinical manifestations, PD and dental caries were often regarded as two independent diseases and studied separately. Nonetheless, the association between PD and dental caries has been debated in recent years, with some studies showing an inverse association and others showing a positive association between these two diseases [21, 22]. However, little was still known about the direction and nature of the association between these two conditions. The overall aim of this study was to conduct a robust critical appraisal of the evidence on the relationship between PD and dental caries, mainly in terms of correlation of the clinical prevalence and the predominant causative organisms between the two diseases.

## Method

### Search strategy

Studies were selected based on the PECOS question, including observational studies in humans with caries/periodontitis (P—persons) in which periodontitis/caries was present (E—exposure) or absent (C—comparison) to observe the prevalence of caries/periodontitis and the relationship of major pathogenic bacteria (O—outcome). Hence, we addressed several key questions: Is there any association between periodontitis and caries? Is it positive or negative and are there any clinical and bacteriological correlation between these two diseases?

The search string considered alternate terms incorporating several relevant key words and Medical Subject Headings (MeSH). The final Boolean search string was: (periodontal diseases∗ OR periodontitis) AND (dental caries* OR caries) **(**Appendix S1**)**. The search string was applied from PubMed and EMBASE databases as well as grey literatures until July 5th 2023 to ensure retrieval of a broad scope of literature.

### Eligibility criteria

Eligible studies were examined by two authors independently. The final selection was verified by a third author, and disagreements were resolved through discussions.

#### Inclusion criteria

Dental caries condition in patients with periodontitis.

- Population: human.
- Exposure: subjects with periodontitis. The confident case definition for periodontitis were defined as periodontal pocket depth (PPD) ≥ 4 mm, clinical attachment loss (CAL) ≥ 3 mm, and community periodontal index (CPI) ≥ 3 [23]; The non-confident case definition was considered as ‘Alveolar bone loss’ without other measurements of PPD/CAL; Unclear diagnostic criteria for periodontitis.
- Non-exposure: subjects without periodontitis (with periodontal health or gingivitis).
- Outcome: the primary outcome was defined as prevalence of dental caries (crown caries or root caries) in individuals with periodontitis, the secondary outcome was mean DMFT (the amount of decayed, missing, filled permanent teeth) or DFR (the amount of decayed, filled root teeth), along with the correlation of pathogenic bacteria related to caries and PD.
- Study design: case–control or cohort studies.

Periodontitis condition in patients with caries.

- Population: human.
- Exposure: subjects with caries, it was defined as DT ≠ 0.
- Non-exposure: subjects without caries.
- Outcome: the primary outcome was defined as prevalence of periodontitis in individuals with caries; the secondary outcome was the correlation of pathogenic bacteria related to caries and periodontitis.
- Study design: case–control or cohort studies.

#### Exclusion criteria

- Studies with groups evaluating periodontitis or caries separately, case reports, review or guidelines, no full text available nor English language used.
- Publications were further excluded if periodontal status was only assessed by tooth loss or gingival appearance.

### Quality assessment

Newcastle–Ottawa Scale (NOS) [24] was employed to evaluate the methodological quality of the included studies, which were defined as moderate or high methodological quality with at least five scores.

### Data extraction and processing

Data extraction conducted by YX.Li and YG.Xiang was based on a standardized, pre-piloted data extraction form. The extracted information included: authors and year, study design, characteristics of the sample (size, age, location, and study group); evaluation method (periodontitis and caries diagnosis), statistical analysis, the primary outcomes ([RR] or [OR], 95% confidence interval (CI) or those providing absent raw numbers are available for crude calculation) and the secondary outcomes (mean DMFT or DFR, and the association and interaction of pathogenic bacteria associated with caries and periodontitis).

### Statistical analysis

The estimates (or adjusted estimates if applicable) and the corresponding 95% confidence interval (CI) between PD and caries were used to calculate the pooled estimates. If no estimates were available in the studies, the numbers of cases (with PD/caries or not) and controls (with PD/caries or not) were used to calculate the pooled estimates. Heterogeneity was evaluated using the Cochrane I^2^ statistic [25]. Descriptive statistics were performed to summarize the evidence retrieved, checking further for study variations in terms of study characteristics and results. For caries condition in patients with PD, subgroups analyses were performed in periodontitis diagnostic criteria, periodontitis severity, type of caries, age and gender. Similarly, for PD risk in patients with caries, the subgroups analyses were performed in age and gender. Funnel plot was generated to assess publication bias for primary outcome by the visual inspection of asymmetry. Rev Man 5.1 was employed to perform all analyses.

## Results

### Literature selection

**Fig. 1:**
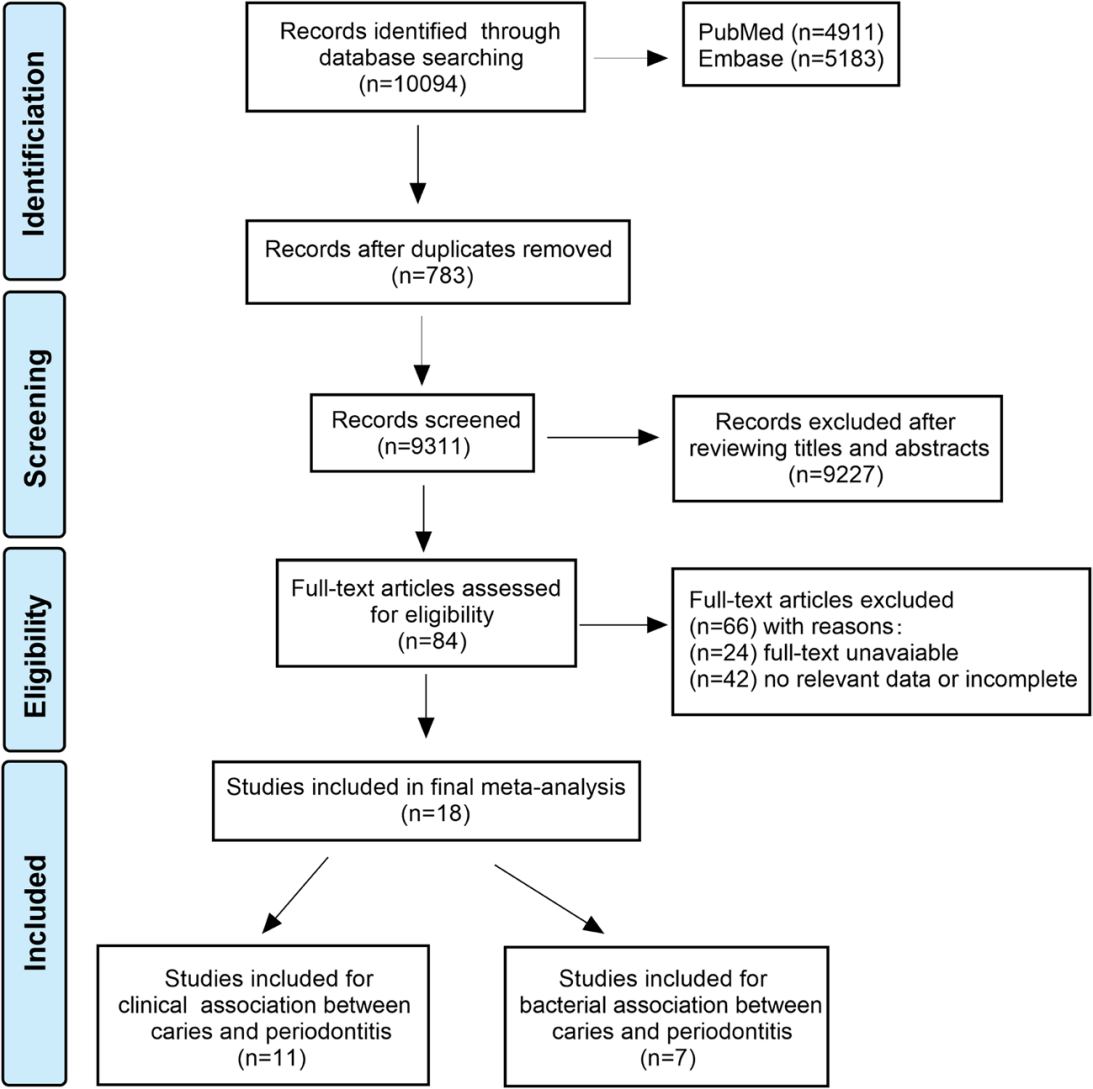
PRISMA flowchart: selection process of studies and results of the literature search for meta-analysis

The literature search process was summarized in Fig. 1. Briefly, 10,094 articles were retrieved by an initial database search, including exclusion of 783 duplications. 9,227 publications were excluded after screening the abstracts. 84 publications were eligible for full text screening. Finally, a total of 18 publications were included for final meta-analysis.

### Study characteristics and quality assessment

All included studies were published between 1994 and 2022. The characteristics of these 18 studies were shown in Table 1, including 10 studies [26–35] in relative to the prevalence of caries in patients with periodontitis, 5 [29–32, 36] containing the prevalence of periodontitis in patients with caries and 7 studies [33, 37–42] covering the association between pathogenic bacteria related to two diseases.

**Table 1:** Characteristic of included studies

Author, year, country	Studies design	Population of study	Periodontal criteria	Caries criteria	Observed effect (OR, RR, etc.…)
María 2021 Spain	Case–control	N = 5130	CPI (0–4); CAL (0-3 mm, 4-6 mm, ≥ 6 mm)	DMFT > 0	Subjects with CAL 0–3 mm (n = 4070), 4–5 mm (n = 685) and ≥ 6 mm (n = 375) groups had a DMFT index of 7.8, 9.6 and 10.5; DFR index was 0.2, 0.5 and 0.8 respectively, with a statistically significant difference (*p* < 0.001). The prevalence of caries was 38.2%, 42.9%, and 47.7%
		Age			In the logistic regression analysis, having CPI ≥ 3, CAL ≥ 4 mm were significantly associated with a higher prevalence of cavitated caries (OR _crude_ = 1.9, 95% CI [1.7–2.1], *p* < 0.001); After adjustment for confounders, having CPI ≥ 3 was significantly associated with a higher prevalence of cavitated caries (OR _adjusted_ = 1.6, 95% CI [1.3–1.8], *p* < 0.001)
		Gender:			
		Female = 2083			
		Male = 3047			
Vehkalahti 1994 Finland	Case–control	N = 4777	PD > 4 mm	DT ≠ 0	Among female, the OR for the association between root caries and moderate periodontitis or severe periodontitis were 2.9 and 3.2 respectively
		Age:			Compared with subjects without periodontitis, the OR were 5.4 and 9.3 for men with 4–6 mm and 6 mm deep pockets
		mean = 46			Overall, periodontitis was associated with root caries OR = 1.3 (0.9–1.7)
Aron 2019 Sweden	Case–control	N = 1577	Alveolar bone loss: healthy: 80% bone remnant; mild to moderate (79–66%); severe: (> 66%)	DT ≠ 0	The prevalence of caries in no-periodontitis patients (n = 988), mild-moderate (n = 492) and severe periodontitis patients (n = 97) were 16%, 19%, 30%
		Age < 75			
Franz 2019 Chile	Case–control	N = 994	CDC-APP	DT ≠ 0	The prevalence of periodontitis (89.3%) was higher in patients with caries (n = 855) than in those without caries (n = 139) (78.4%), *P* < 0.05
		Age: 35–44	CAL > 3 mm		
		Gender:	PD > 4 mm		
		Female = 558			The prevalence of caries in patients with periodontitis (n = 873) or without periodontitis (n = 121) was 89.3% and 10.6%
		Male = 436			The prevalence of caries in moderate (n = 498) and severe (n = 251) periodontitis patients was 58.2% and 29.3%
Belstrøm 2014 Denmark	Case–control	N = 586 Patients saliva samples, including periodontitis group (n = 139) and randomized control cohort (n = 447), 5% of this group also had periodontitis	BOP > 25%, (≥ 2 teeth) CAL > 4 mm and PD > 6 mm	DT ≠ 0	The prevalence of periodontitis was 6% in patients with caries (n = 417) and 4% in those without caries (n = 30)
					The prevalence of caries in patients with periodontitis (n = 426) was 6.57%and 9.5% in those without periodontitis (n = 21)
Amare 2022 Ethiopia	Case–control	N = 443	PD > 3 mm	DT ≠ 0	The prevalence of periodontitis in patients with (n = 184) and without (n = 259) caries was 34.8% vs 22.4%
		Age:7–30			
		Gender:			The prevalence of caries in patients with (n = 122) and without (n = 321) periodontitis was 34.8% vs 65.2%
		Female = 206			Multiple regression showed that caries had an effect on the risk of periodontitis (OR = 1.85, 95% CI: 1.21–2.82)
		Male = 237			
Mattila 2010 Finland	Case–control	N = 5255	PD > 4 mm	DT ≠ 0	The prevalence of caries in patients with (n = 68) and without (n = 44) periodontitis was 33% vs 23%, which was higher in severe periodontitis (44%)
		Age: > 30			The prevalence of periodontitis in patients with (n = 1524) and without (n = 3731) caries was 31% vs 16%
		Gender:			
		Female = 2782			
		Male = 2473			
Hani 2011 Saudi Arabia	Case–control	N = 112	Alveolar bone loss	DT ≠ 0	The prevalence of coronal caries in patients with (n = 68) and without (n = 44) periodontitis was 94% vs 93%
					The prevalence of root caries in patients with (n = 68) and without (n = 44) periodontitis was 29% vs 5%
Lina 2020 England	Case–control	N = 4738	16-34 s: diagnosed with PD > 35 s: diagnosed with CAL	DT ≠ 0	Adults with PD ≥ 4 mm had a 1.03 rate ratio (RR) for coronal caries(*P* < 0.01) and 1.23 rate ratio (RR) for root caries (*P* < 0.001)
		Age > 35			
Li 2021 China	Case–control	N = 35-44 s 4407 65-74 s 4117	CAL	DT ≠ 0	Patients with periodontitis in the 35-44 s were significantly associated with caries DFT, with type B: OR = 1.21 (1.17–1.25) and type C: OR = 1.40 (1.24–1.56)
				A: crown caries	
				B: mixed-type caries	Patients with periodontitis in the 65-74 s were significantly associated with caries DFT, with type C: OR = 1.28 (1.21–1.35)
				C: root caries	The prevalence of caries with moderate(n = 2552) or severe (n = 2198) periodontitis were 66.2% and 82.5%
Gürlek 2021 Turkey	Case–control	N = 100 (including 50 women of childbearing age and 50 infertile women) Age: 21-39 s	PD > 4 mm +	Combined clinical and imaging diagnosis DMFT	Both in all subjects and only in infertile women, caries was associated with periodontitis, the former OR = 1.39(1.17–1.65) and the latter OR = 1.40(1.07–1.82)
			CAL > 3 mm		
Shrestha 2016 Nepal	Case–control	N = 100	CPI	DMFT	The DMFT indices of periodontitis patients with CPI classification (0–2) and (3–4) were 4.70 ± 2.80; 4.09 ± 3.61
					There were significant differences in number of teeth present between the control group and periodontitis group (OR _crude_ = 1.29, 95% CI: 1.05–1.58, *P* = 0.014); (OR _adjusted_ = 1.55, 95% CI: 1.15–2.09, *P* = 0.004)
		Age: 27-58 s			
Robert 2019 Canda	Case–control	N = 94	PD/CAL	DM_3_FS: a missing tooth counts as 3 missing surfaces	Significant positive associations were found between the periodontal disease severity (CDC-AAP) and the DMFS (OR _adjusted_ = 1.03; 95% CI: 1.01–1.05) and DS indices (OR _adjusted_ = 1.18; 95% CI: 1.05–1.32) as well as between the tertiles of percentage of sites with CAL ≥ 3 mm and DMFS (OR _adjusted_ = 1.03; 95% CI: 1.00–1.05) and DS indices (OR _adjusted_ = 1.12; 95% CI: 1.00–1.25)
		Age > 30 s			A significant positive association was also found between oral levels of F. nucleatum and S. mutans (OR _adjusted_ = 6.03; 95% CI: 1.55)
Kozlovsky 2015 Tel Aviv	Case–control	N = 30 (LAgP11 + GAgP19)	PD	DFS	DFS index was 10.62 ± 6.8, 7.5 ± 6.5 in GAgP and LAgP patients
		Age:			The number of patients with GAgP with high, medium and low (10^5^-10^3^C FU/ml) levels of S. m was 3/2/11
		LAgP:23.5 s			The number of LAgP patients with high, medium and low (10^5^-10^3^ CFU/m l) levels of S. m was 3/2/4
		GAgP:30.5 s			
Van 2001 The Netherlands	Case–control	N = 154	PD	The prevalence and rates of streptococcus mutans	The prevalence of Streptococcus mutans in the saliva of periodontitis patients in the untreated group vs. the post periodontal surgery group increased from 82 to 94%
					The mean proportions of Streptococcus pyogenes in the saliva of periodontitis patients in the untreated group vs. periodontal maintenance group were 6.65% and 1.86%, *P* = 0.005. After periodontal surgery, it reporter 2,51%, *P* = 0.041
Gizani 1999 Belgium	Case–control	N = 10	PD	Levels of Streptococcus pyogenes and Lactobacillus	Compared to the baseline Streptococcus pyogenes level of 2.5*10^5^ ± 4.3*10^5^, the level of Streptococcus pyogenes on the dorsum of the tongue increased significantly at 4/8 months to 4.8*10^5^ ± 1.0*10^6^ and 3.1*10^6^ ± 6.8*10^6^ after periodontal treatment
		Age = 23-66 s			In saliva, Streptococcus mutans increased significantly to 2.4*10^4^ ± 5.1*10^4^ at 8 months; in dental plaque, Lactobacillus content increased significantly at 8 months
		Female = 4			
		Male = 6			
Iwano 2009 Japan	Case–control	N = 40	CPI	DT ≠ 0	Salivary levels of Streptococcus pyogenes increased significantly in 10 patients with periodontitis before and after periodontal treatment, *P* < 0.05
		Age = 23-78 s			
		Gender:			
		Female = 23			
		Male = 17			
Yasuhiko 2006 Japan	Case–control	N = 368	PD	Levels of Streptococcus pyogenes and Lactobacillus	Values > 4 mm of attachment loss (rAL4) and for average attachment loss (a AL) of sites measured were significantly higher in subjects with LB than those without Multiple regression analysis also showed a correlation between a AL and rAL4 values with the presence of LB (a AL p ¼ 0.003; rAL4 p ¼ 0.002)
		Age = 75 s	CAL		Further, multiple regression analysis of interacting factors regarding decayed root surfaces showed that LB carriers had a greater incidence of decayed root surface caries (p ¼ = 0.003), while MS and LB levels were correlated to the number of decayed root surfaces (LB p ¼ 0.010; MS p ¼ 0.026)

Note: *N* number, *DMFT* the number of decayed missing and filled teeth, *DFR* the number of decayed and filled root teeth, *PD* periodontal pocket, *CAL* clinical attachment loss, *AL* attachment loss, *CPI* community periodontal index, *LAgP* localized aggressive periodontitis, *GAgP* generalized aggressive periodontitis, *MS* mutans streptococci, *LB* lactobacilli, *rAL4* rate of sites with > 4 mm of attachment loss, *aAL* average attachment loss, *OR* odds ratio, *RR* relative risk

Study quality for observational studies assessed by the Newcastle–Ottawa scale varied across the studies, ranging from a score of 5/9 to 8/9 **(**Appendix S2**)**. The assessment revealed several potential sources of bias including the representativeness of the cases and lack of adjustment for potential confounders.

### Dental caries risk in patients with periodontitis

**Fig. 2:**
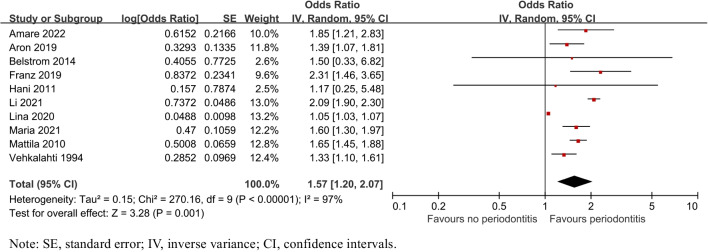
Forest plot for caries risk of patients with periodontitis

To assess the risk of having caries in individuals with periodontitis, all the 10 relative studies [26–35] were included. The pooled results showed that the risk of having caries was associated with periodontitis (OR = 1.57, 95% CI: 1.20–2.07, P = 0.001) in Fig. 2.

#### Subgroup analysis

##### Definition of periodontitis

**Fig. 3:**
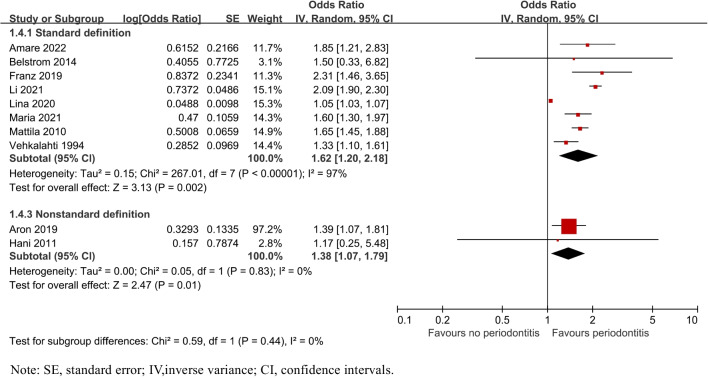
Forest plot by definition of periodontitis subgroup analysis for caries risk of patients with periodontitis

Subgroup analysis by the definition of periodontitis indicated that 8 studies [26, 27, 29–32, 34, 35] with standard definition of periodontitis confirmed higher odds of caries (OR = 1.62, 95% CI: 1.20–2.18; P = 0.002) as well as 2 studies [28, 33] with a non-standard definition of periodontitis. (OR = 1.38, 95% CI: 1.07–1.79; P = 0.01, Fig. 3).

##### Periodontitis severity

**Fig. 4:**
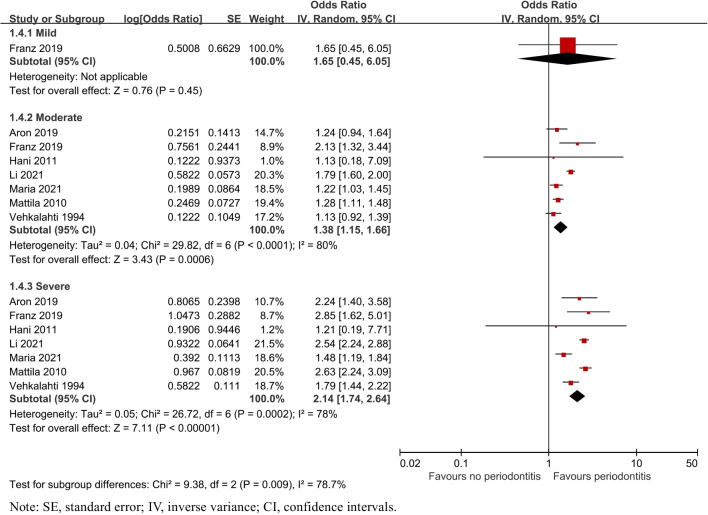
Forest plot by severity of periodontitis subgroup analysis for caries risk of patients with periodontitis

Subgroup analysis by severity of periodontitis demonstrated that caries was significantly associated with moderate (OR = 1.38, 95% CI: 1.15–1.66, P = 0.0006) and severe periodontitis (OR = 2.14, 95% CI: 1.74–2.64, P = 0.0002). One study [29] reported the risk of mild periodontitis with caries (OR = 1.65, 95% CI: 0.45–6.05) in Fig. 4.

##### Different surfaces of caries

**Fig. 5:**
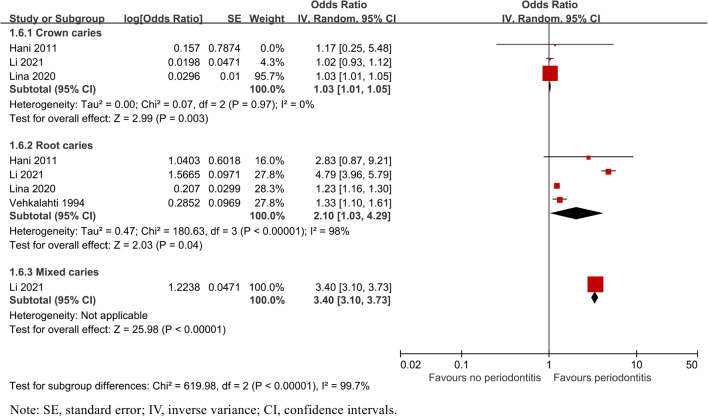
Forest plot by different surfaces of caries subgroup analysis for caries risk of patients with periodontitis

Subgroup analysis by different surfaces of caries showed that the risk of the site where caries occurs varied in crown caries (OR = 1.03, 95% CI: 1.01–1.05, P = 0.003) and root caries (OR = 2.10, 95% CI: 1.03–4.29, P = 0.04). Especially, an increased risk of mixed-type caries defined as covering crown and root surfaces in one study [35] was reported (OR = 3.40, 95% CI: 3.10–3.73, *P* < 0.00001, Fig. 5).

##### Age and gender

**Fig. 6:**
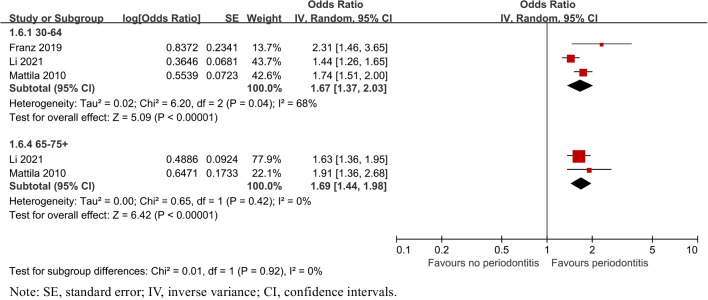
Forest plot by age subgroup analysis for caries risk of patients with periodontitis

**Fig. 7:**
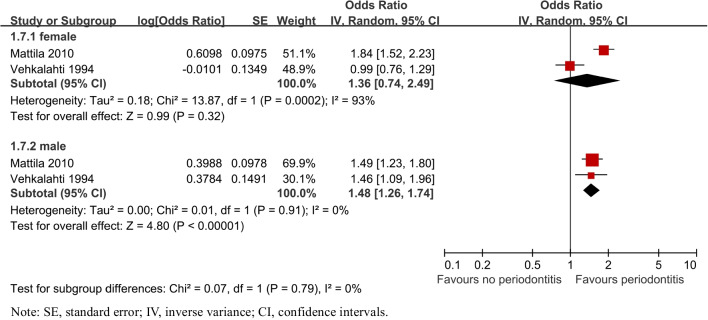
Forest plot by gender subgroup analysis for caries risk of patients with periodontitis

Additionally, subgroup analyses were also performed by gender and age in Figs. 6 and 7. The risk of caries was both higher in 30 to 64 years old (OR = 1.67, 95% CI: 1.37–2.03, *P* < 0.00001) and 65 to over 75(OR = 1.69, 95% CI: 1.44–1.98, *P* < 0.00001). Two studies [27, 32] reported the risk of dental caries between male and female, respectively, which varied in male (OR = 1.48, 95% CI: 1.26–1.74, *P* < 0.00001) and female (OR = 1.36, 95% CI: 0.74–2.49, P = 0.32).

##### OR adjusted or not

**Fig. 8:**
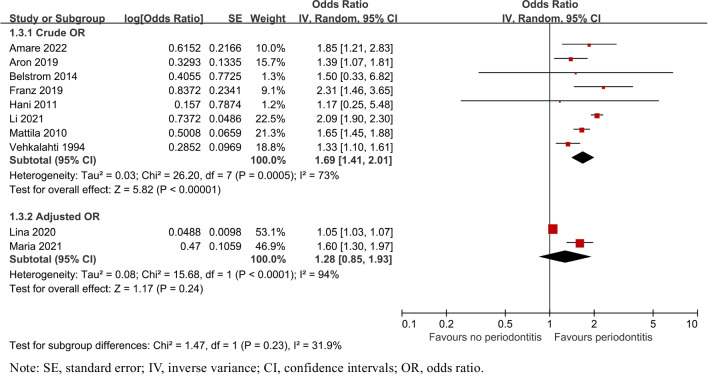
Forest plot by OR adjusted or not subgroup analysis for caries risk of patients with periodontitis

Subgroup analysis by OR adjusted or not showed that 2 studies [26, 34] reported adjusted ORs, where the precision of the estimate based on the confidence interval bounds was not significant (OR = 1.28, 95% CI: 0.85–1.93, P = 0.24). 8 studies [27–33, 35] provided absent raw numbers available for crude calculation (OR = 1.69, 95% CI: 1.41–2.01, P = 0.0005, Fig. 8).

#### Mean DMFT or DFR

Four studies [26, 33, 34, 43] reported mean DMFT or DFR in patients with periodontitis. One study [37] indicated significant positive associations between the periodontal disease severity (CDC-AAP) [23] and the amount of decayed, missing and filled teeth surfaces (DMFS) (OR _adjusted_ = 1.03, 95% CI: 1.01–1.05) and the decayed surfaces (DS) indices (OR _adjusted_ = 1.18, 95% CI: 1.05–1.32) as well as between the percentage of sites with CAL ≥ 3 mm and DMFS (OR _adjusted_ = 1.03, 95% CI: 1.00–1.05) and DS indices (OR _adjusted_ = 1.12, 95% CI: 1.00–1.25). Overall meta-analysis showed Patients with periodontitis exhibited higher DMFT [weighted mean difference (WMD) = 0.87, 95% CI: -0.03–1.76, P = 0.06] and DFR (WMD = 1.13, 95% CI: 0.48–1.78, P = 0.0007) when compared with patients without periodontitis (Appendix S4).

### Periodontitis risk in patients with caries

**Fig. 9:**
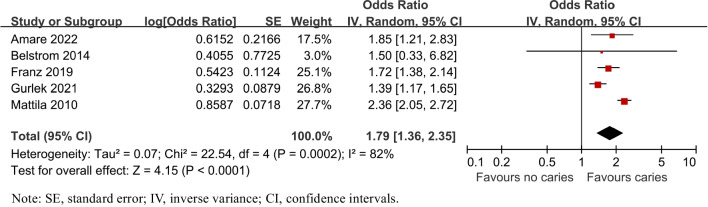
Forest plot for periodontitis risk of patients with caries

**Fig. 10:**
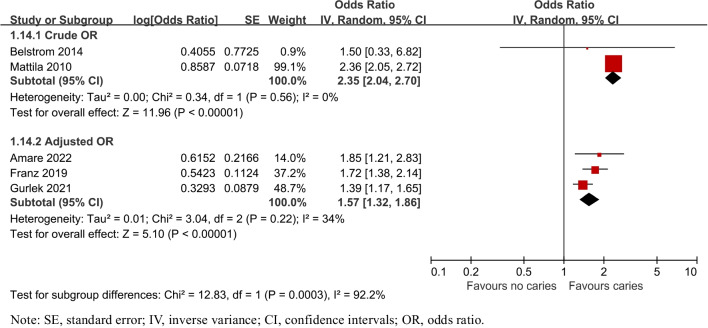
Forest plot by OR adjusted or not subgroup analysis for periodontitis risk of patients with caries

To assess the risk of having periodontitis in individuals with caries, all the 5 relative studies [29–32, 36] were included. Statistically significant heterogeneity was confirmed with I^2^ test (I^2^ = 82%, P = 0.0002). The pooled results in Fig. 9 showed that the risk of periodontitis was associated with caries (OR = 1.79, 95% CI: 1.36–2.35, P = 0.0002), both for crude ORs (2.35, 95% CI: 2.04–2.70, *P* < 0.00001) and adjusted ORs (1.57, 95%CI: 1.32–1.86, *P* < 0.00001, Fig. 10). Other subgroups, such as types of caries, severity of periodontitis, age and gender, were not analyzed due to limited data.

### Bacterial association between periodontitis and caries

Seven studies [33, 37–42] were included as reporting the correlation between the dominant pathogenic bacteria associated with PD and caries. An increased risk of detectable high level (> 10^4^ CFU/ml) of P. gingivalis was reported in the saliva when PD occurred (OR = 3.38, 95% CI: 1.46–7.83, P = 0.005), while S. mutans, one of the main pathogens of caries, showing a declining risk in PD (OR = 0.82, 95% CI: 0.44–1.51), although no significant difference (P = 0.470, Appendix S5). Three studies [37, 38, 40] reported the relationship between levels of bacteria in saliva samples and loss of attachment. One of them [40], showed that patients with high Lactobacillus (> 10^5^ CFU/ml) in saliva had significantly higher average attachment loss (P = 0.014) and rate of sites with > 4 mm of attachment loss (P = 0.014). S. mutans followed the same trend as Lactobacillus. Similarly, another study by Robert et al. [37] showed that high levels of S. mutans (> 10^5^ CFU/ml) in saliva were negatively correlated with a decrease in the sites of loss of attachment > 3 mm, OR _adjusted_ = 0.74 (0.35–1.60), P = 0.447. Besides, this study reported a significant positive correlation between levels of F. nucleatum and S. mutans [OR _adjusted_ = 6.03 (1.55–23.45), P = 0.009] as well as Actinobacillus [OR _adjusted_ = 2.39 (1.00–5.71), P = 0.051]. What’s more, one study [38] reported the relationship of pathogenic bacteria in the saliva of patients with aggressive periodontitis, which found that the level of Actinobacillus in saliva was positively correlated with the proportion of deep periodontal pockets (> 7 mm), P = 0.03, and negatively correlated with the level of S. mutans. Among them, Actinobacillus was detectable in 25 out of 30 aggressive periodontitis (AgP) patients, 15 of which showed low levels of S. mutans in their saliva (< 10^3^ CFU/ml), indicating a negative correlation (OR = 0.08, 95% CI: 0.01–0.85, P = 0.005) between them. Moreover, the level of Actinobacillus in saliva of patients with low level of S. mutans (< 10^3^ CFU/ml) was significantly higher than that of patients with high level of S. mutans (> 10^5^ CFU/ml) and medium level of S. mutans (10^3^–10^5^ CFU/ml), P = 0.009. Three studies [39, 41, 42] reported the effect of periodontal treatment on periodontitis and caries bacteria. As summarized in Appendix S6, compared to pre-treatment, the prevalence of high levels (> 10^4^ CFU/ml) of S. mutans increased after scaling and root planning (OR = 1.54, 95% CI: 0.47–5.02), but there was no statistically significant difference. (P = 0.470). Nevertheless, the mean level of S. mutans was significantly higher (*P* < 0.05) after periodontal therapy. Another study [42] assessed levels of saliva and supragingival plaque during periodontal treatment. The amount of S. mutans in saliva increased significantly at 8th month (*P* < 0.05). For supragingival plaque, the CFUs of S. mutans remained basically unchanged, but the frequency of detection increased from 4/10 to 6/10. lactobacilli decreased significantly at 8th month compared to preoperative (*P* < 0.05).

### Publication bias

Study publication bias was examined using funnel plot for caries risk in patients with PD in Appendix S7. Visual assessment of the Funnel plot revealed studies was displayed nearly symmetrical appearance, indicating no significant bias.

## Discussion

This systematic review supported a positive association between PD and caries. In terms of caries condition in patients with PD, patients with moderate to severe periodontitis had a higher (57%) chance of having caries compared to non-periodontitis patients. In addition, a positive linear relationship was observed, confirming that the more severe the periodontitis was, the higher the likelihood of having caries. Based on the classification of caries between coronal and root caries, the study found that patients with periodontitis had an elevated likelihood of having root caries.

This systematic review also attempted for the first time to provide estimates of the mean DMFT and DFR in periodontitis patients. Interestingly, 50% of the included studies reported an increased DMFT in patients with periodontitis. However, all of the included studies showed an elevated mean DFR in patients with periodontitis. The association between periodontitis and root caries observed in this study was in agreement with previous studies [44], in which periodontitis was correlated with the prevalence of root caries.

In terms of the periodontal condition of patients with caries, patients also had a higher (79%) risk of having periodontitis compared to controls. This result was in line with the findings of the study [29], which showed that people with three or more untreated carious lesions were more likely to develop periodontal disease. The explanation for this phenomenon may be that untreated carious lesions may increase plaque retention and susceptibility to periodontal disease [45]. This was observed in a 3-year longitudinal study in which untreated carious lesions in adolescents had a significant negative impact on periodontal health [46].

The positive correlation between the two diseases can be explained by common risk factors, including oral hygiene practices [47], the presence of dental biofilms, lifestyle habits [48] and social factors [49]. Dental plaque biofilms, which are the initiators of caries and periodontitis, constituted distinct polymicrobial communities. With dynamic changes in community composition, microbial metabolism and by-products that triggered host immune responses can cause changes in the local environment, thereby affecting the growth or colonization of single or multiple microbial populations, promoting the growth or over-activity of microorganisms associated with ecological maladies, and thus influencing the development of the disease [17].

In the same oral environment, the positive correlation between caries and periodontitis was found to be closely related to its oral hygiene environment. Jiang et al. [50] analyzed the periodontal status of 86 pairs of carious teeth and found that the degree of gingival recession, the depth of periodontal probing, and calculus index were significantly higher in the carious group than in the no-caries group, regardless of whether they suffered only from crown caries or crown-root caries. This may be due to the fact that caries tends to occur on tooth surfaces that are not easily cleaned and where food debris tends to linger, and these areas are often where plaque tends to accumulate. As caries progressed, pain from food embedded in the cavity or temperature stimulation can occur, leading patients to unconsciously avoid these sensitive areas when brushing and rinsing, which in turn made plaque and calculus more likely to accumulate. This provided a favorable environment for the survival of periodontal pathogenic bacteria, leading to the development of periodontitis. Similarly, periodontitis caused the gums to recede, leading to root exposure. Due to the temperature sensitivity of the exposed roots, the teeth on the affected side were disused and prone to food debris accumulation. At the same time, the lack of periodontal tissue allowed for increased loss of teeth in severe periodontitis, resulting in larger gaps between the teeth, which made it easier for food to become embedded. The environment of the affected side favored the growth and metabolism of cariogenic bacteria to form caries. Therefore, the close relationship between oral hygiene, plaque biofilm and the two diseases can be used to support the positive correlation between caries and periodontitis.

In particular, one of the explanations for the strong association between periodontitis and root caries was that the exposure of cementum/dentine due to recession caused by PD may provide a new substrate for bacterial adhesion, and favor colonization of Gram-negative proteolytic species that can degrade the endogenous collagen and other organic components of these tissues [51]. Furthermore, bacteria metabolized sugar into organic acids, which initiated root surface demineralization by removing calcium and phosphate ions from surface apatite crystals. For enamel, this process took place as the pH reached the critical value of 5.5; however, pH 6.4 was sufficient for cementum and dentin demineralization, due to their lower degree of mineralization [52] as well as limited amount of fluoride and poorer caries resistance than enamel. Traditional periodontal treatment reducing the resistance to caries may be another reason that mechanical removal of plaque, scaling and root planning affected the outer part of the root surface and exposed cementum and dentin surfaces with low caries resistance [42]. In addition, gingival recession enables saliva to access the root surface, which is believed to be one of the most important host factors and an essential mediator controlling the speed and direction of the cariogenic pathway. Saliva was shown to neutralize the pH level on the root surface by a salivary buffering action and changed nutritional conditions, affecting the environment and nutrients of bacteria [13].

Apart from the correlation at the clinical level, this study also summarized the correlation between periodontitis and caries-related causative organisms. The results showed that patients with periodontitis had decreased salivary levels of S. mutans, one of the main caries-causing organisms, compared to those without periodontitis. Besides, several studies demonstrated that patients with high salivary levels of S. mutans (> 10^5^ CFU/ml) had reduced mean attachment loss and the number of sites with attachment loss > 4 mm. These results could be explained by the fact that periodontitis mediates changes in the microecological community, leading to increased inflammation and alterations in the microbiological composition of oral biofilms [53]. Some of these reports suggested an antagonistic relationship between bacteria associated with the oral microbiota of both diseases. Numerous clinical trials have confirmed a negative correlation between P. gingivalis and S. mutans both in subgingival plaque and saliva before and after systemic treatment of periodontal disease patients [39]. Different degrees of antagonism were found between the two bacteria when they were co-cultured, in which P. gingivalis was found to interfere with the quorum sensing (QS) system of S. mutans, thereby diminishing its biofilm-forming ability, acid tolerance, and bacteriocin-producing ability [54]. However, some studies [37] have also shown a positive correlation between other periodontal pathogens such as F. nucleatum, Actinobacillus and S. mutans, which may be mediated by the co-aggregation of F. nucleatum [55] and the high genetic variability of Actinobacillus strain [56]. Therefore, further long-term cohort and elementary research on the bacterial association between periodontitis and caries should be carried out, in order to better understand the pathogenetic relationship between the two diseases, which can facilitate innovative strategies for the prevention and treatment of these two diseases with highly prevalence.

In this systematic review, for the first time, we conducted the correlation between PD and dental caries at the clinical and bacterial levels. Interestingly, either periodontitis or caries as independent variable, the correlation between the two diseases was positive and significant. In addition, the pooled results of all the included studies indicated that patients with periodontitis had a higher DMFT/DFR index. However, at the bacterial level, there seemed to be a negative correlation between the levels of the main dominant pathogenic bacteria related to two diseases in the same microecological environment. Studies have shown that environmental acidification was the main cause of phenotypic and genotypic changes in the microbiota during the development of dental caries [57], with S. mutans and Lactobacillus being identified as specific caries pathogens [11]. However, S. mutans was not only present at high levels in the early stages of dental caries, but also in healthy humans, even at very low levels [58, 59]. This seemingly contradictory results seemed to explain the trend related to the clinical and bacterial dimensions in this study, where both caries and periodontitis were based on a multiple microbiological community and a variety of other factors influencing the course of the disease, rather than being determined by a single or a select few bacterial species [4, 60]. In other words, dysbiosis contributed to diseases means an alteration in the abundance or influence of individual species within the polymicrobial community, relative to their abundance or influence in health. Whereas dysbiosis can lead to destructive periodontal inflammation, the reverse is also true. In this respect, inflammatory tissue breakdown products such as degraded collagen and heme-containing compounds are released into the gingival crevicular fluid. In the gingival crevice/pocket, these inflammatory spoils can be used as nutrients to fuel the selective expansion of a subset of bacterial species like proteolytic and asaccharolytic pathobionts, thereby exacerbating the imbalance of the dysbiosis [61–63]. In oral ecosystems, the proximity of microorganisms promoted a series of biochemical interactions that may be favorable to one organism and antagonistic to the other. Therefore, further studies combining clinical and microbiological measures of both these diseases are necessary for better understanding of the possible relationship between these two diseases which may lead to improved management and prevention.

## Strengths and weaknesses

This systematic review was designed to comprehensively investigate the trends between caries and periodontitis. As a result, the bidirectional relationship between dental caries and periodontitis both in clinical and bacterial levels was first analyzed, and more precise conclusions through subgroup analysis were drawn. Meanwhile, this study analyzed the DMFT/DFR index of patients with periodontitis for the first time, and especially the strong correlation with root caries was important for the treatment and prevention of patients with periodontitis. For example, the inclusion of caries prevention treatment in periodontal sequence therapy or the adjustment of the follow-up time for patients with severe periodontitis was important in PD therapy. Furthermore, the correlation between two diseases at the bacterial level suggested that symbiosis or antagonism between one or several pathogenic bacteria may not directly determine the progression of the disease, but may also cause the disease through affecting the oral microecological balance, bacterial quorum sensing (QS) mechanism and host-community interaction. Therefore, further research in identifying the interplay between pathogens in each individual and their relative contribution on each other is needed. However, a number of limitations should be highlighted starting with the limited value of this systematic reviews of observational studies for ascertaining causality. Moreover, the definition of periodontitis in several studies seemed a slight discrepancy as well as bias in clinical examination, which may lead to selection bias. Not all included studies were adjusted for potential confounders such as diet, smoking habit and socio-economic level. The true value could be distorted by these confounding factors. Further intervention studies and long-term are needed to establish this causal relationship. What’s more, Due to the limited number of included studies and subjects, no further subgroup analysis between sexes or ages were performed.

## Conclusions

This study demonstrated that both of two disorders were linked by two-way relationships. Especially, the risk of dental caries was increased by the severity of periodontitis. The presence of periodontitis was also associated with an increased DMFT and DFR. At the bacterial level, however, the pathogens associated with two disorders were negatively correlated. Our findings highlighted the necessity to improve caries prevention during periodontal treatment. Longer and larger studies are needed however to determine whether periodontal treatment with concomitant caries management facilitates disease therapy and prognosis, and how intrinsic bacterial interactions or immune regulation affect the disease, ultimately resulting in reduced morbidity.

### Supplementary Information

Below is the link to the electronic supplementary material.

Supplementary file1 (DOCX 344 KB)

## Data Availability

The authors confirmed that the data supporting the findings of this study were available in Table 1 of this manuscript and supplementary materials.
