# Supplementary material for: Association between periodontitis and dental caries: a systematic review and meta-analysis
Source: Clin Oral Investig. 2024 May 10;28(6):306. doi: 10.1007/s00784-024-05687-2 (PMC11087323; doi:10.1007/s00784-024-05687-2)
Supplement: Supplementary file 1 — Supplementary file1 (DOCX 344 KB) [file 784_2024_5687_MOESM1_ESM.docx]

**Appendix 1. Search Strategy**

**Appendix 2. Risk of bias assessment**

**Appendix 3. Periodontitis effect on mean DMFT/DFR**

Note: DMFT, the number of decayed, missing and filled teeth; DFR, the number of decayed and filled root teeth; SD, standard deviation; M, moderate periodontitis; S, severe periodontitis.

**Appendix 4. Summary Forrest plot for change in mean DMFT/DFR in patients with periodontitis**

Note: The graph above showed mean DMFT and the below showed mean DFR for patients with periodontitis; SD, standard deviation; IV, inverse variance; CI, confidence intervals.

**Appendix 5**. **Pooled risk estimates between oral bacteria and periodontitis**

Note: N, number of subjects; CFU: Colony-Forming Units; OR: odds ratio; SD, standard deviation; CI, confidence intervals.

**Appendix 6**. **Pooled risk estimates between oral bacteria and periodontitis treatment**

Note: N, number of subjects; CFU: Colony-Forming Units; OR: odds ratio; SD, standard deviation; CI, confidence intervals.

**Appendix 7. Funnel plot for caries risk in patients with periodontitis**

Note: SE, standard errors; OR, odds ratio.

**Appendix 1. Search Strategy**

| **PubMed** | ((("Periodontitis"[Mesh])OR(Periodontitides[Title/Abstract]))OR(((((("Periodontal Diseases"[Mesh])OR(Parodontosis[Title/Abstract]))OR(Parodontoses[Title/Abstract])))OR(Disease,Periodontal[Title/Abstract]))OR(Diseases,periodontal[Title/Abstract])))AND(((((((((((("Dental Caries"[Mesh])OR(Dental Cavity[Title/Abstract]))OR(Dental Decay[Title/Abstract]))OR(Dental Cavities[Title/Abstract]))OR(Cavities,Dental[Title/Abstract]))OR(Cavity,Dental[Title/Abstract]))OR(Decay,Dental[Title/Abstract]))OR(Carious Lesions[Title/Abstract]))OR(Carious Lesion[Title/Abstract]))OR(Carious Dentin[Title/Abstract]))OR(Carious Dentins[Title/Abstract]))OR((("Root Caries"[Mesh])OR(Caries,Root[Title/Abstract]))OR(Caries,Cervical[Title/Abstract]))) Filters: English |
| --- | --- |
| **Embase** | ('parodontitis' OR 'peridontitis' OR 'periodontitis' OR 'periodontal disease' OR 'dental loss' OR 'parodontal disease' OR 'parodontium disease' OR 'parodontive tissue disease' OR 'peridontal disease' OR 'peridontal tissue disease' OR 'peridontium disease' OR 'periodontal attachment loss' OR 'periodontal diseases' OR 'periodontal infection' OR 'periodontium disease' OR 'tooth loss') AND 'dental caries' OR 'caries' OR 'caries, dental' OR 'carious dentine' OR 'carious teeth' OR 'dental decay' OR 'root caries' OR 'tooth caries' OR 'tooth decay' OR 'dental caries' |

**Appendix 2. Risk of bias assessment**

| **Study**  **(first author)** | **Study**  **Design** | **Selection** | | | |  | **Comparability** |  | **Exposure** | | | **Total Number of stars (maximum 9)** |
| --- | --- | --- | --- | --- | --- | --- | --- | --- | --- | --- | --- | --- |
|  |  | **Case definition adequate?** | **Representativeness of the cases** | **Selection of controls** | **Definition of controls** |  | **Based on**  **design and analysis** |  | **Ascertainment of exposure** | **Same method for ascertainment for cases and controls** | **Non-Response rate** |  |
| María 2021 | Case-control | * | * | * | * |  | * |  | * | * | * | 8 |
| Vehkalahti1994 | Case-control | * | * | * | * |  | * |  | * | * | * | 8 |
| Aron 2019 | Case-control | * | * | * | * |  |  |  | * | * | * | 7 |
| Franz 2019 | Case-control | * | * | * | * |  | * |  | * | * | * | 8 |
| Belstrøm 2014 | Case-control | * |  | * | * |  |  |  | * | * |  | 5 |
| Amare 2022 | Case-control | * |  | * | * |  | * |  | * | * | * | 7 |
| Mattila 2010 | Case-control | * | * | * | * |  | * |  | * | * | * | 8 |
| Hani 2011 | Case-control | * |  | * | * |  |  |  | * | * | * | 6 |
| Lina 2020 | Case-control | * | * | * | * |  | * |  | * | * | * | 8 |
| Li 2021 | Case-control | * | * | * | * |  | * |  | * | * | * | 8 |
| Gürlek 2021 | Case-control | * | * | * | * |  |  |  | * | * | * | 7 |
| Yasuhiko 2006 | Case-control | * |  | * |  |  |  |  | * | * | * | 5 |
| Kozlovsky 2015 | Case-control | * |  | * |  |  |  |  | * | * | * | 5 |
| Robert 2019 | Case-control | * | * | * | * |  | * |  | * | * | * | 8 |
| Shrestha 2016 | Case-control | * |  | * | * |  |  |  | * | * | * | 6 |
| Van 2001 | Case-control | * | * | * | * |  |  |  | * | * | * | 7 |
| Gizani 1999 | Case-control | * | * | * |  |  |  |  | * | * | * | 6 |
| Iwano 2009 | Case-control | * | * | * |  |  |  |  | * | * | * | 6 |

**Appendix 3**. **Periodontitis effect on mean DMFT/DFR**

| **Author/year** | **Periodontitis** | | |  | **No-Periodontitis** | | |  | **P values** | |
| --- | --- | --- | --- | --- | --- | --- | --- | --- | --- | --- |
|  | **N** | **DMFT±SD** | **DFR±SD** |  | **N** | **DMFT±SD** | **DFR±SD** |  |  |  |
| María 2021 | 1060 | M: 9.6(9.2-10)  S: 10.5(9.8-11.1) | M: 0.5(0.4-0.6)  S: 0.8(0.6-1.0) |  | 4070 | 7.8(7.6-7.9) | 0.2(0.2-0.2) |  | <0.001 | <0.001 |
| Hani 2011 | 68 | M: 4.3±4  S: 3.3±4 | M: 0.5±1  S: 1.1±2 |  | 44 | 4.6±4 | 0.4±2 |  |  |  |
| Lina 2020 | 2524 | 17(16.7-17.31) | 10.04(9.73-10.36) |  | 2214 | 15.49(15.18-15.8) | 7.65(7.32-7.98) |  |  |  |
| Shrestha 2020 | 47 | 4.09±3.61 | NA |  | 53 | 4.70±2.80 | NA |  | 0.245 |  |

Note: DMFT, the number of decayed, missing and filled teeth; DFR, the number of decayed and filled root teeth; SD, standard deviation; M, moderate periodontitis; S, severe periodontitis.

**Appendix 4. Summary Forrest plot for change in mean DMFT/DFR in patients with periodontitis**

**
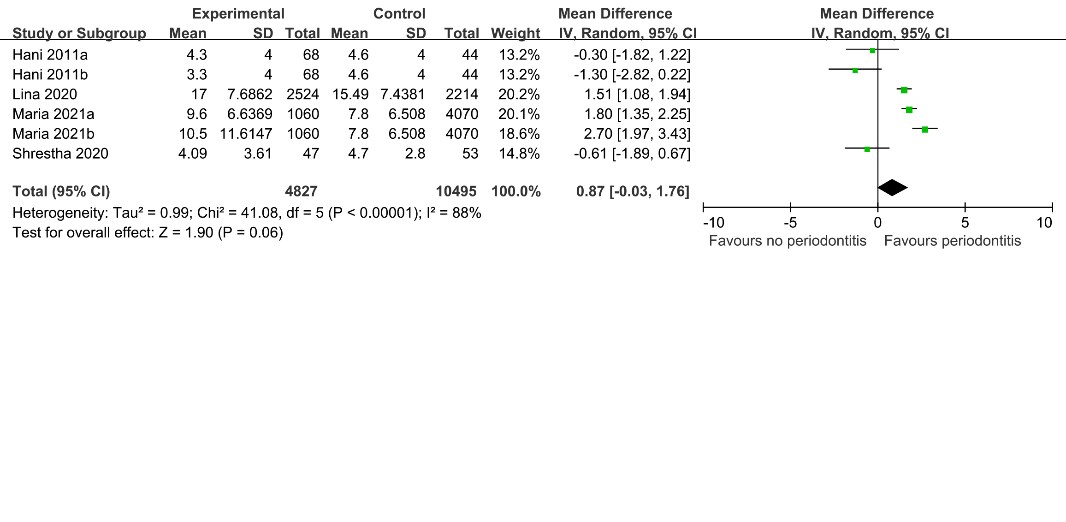

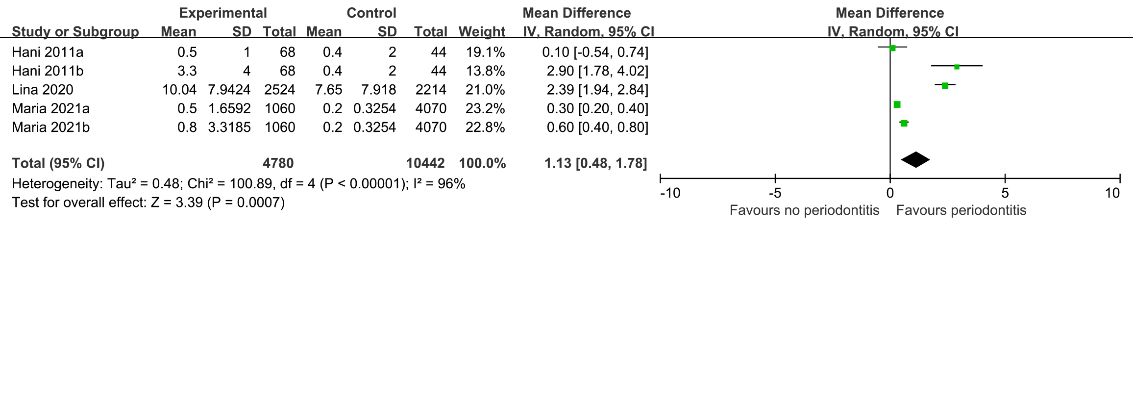
**

Note: The graph above showed mean DMFT and the below showed mean DFR for patients with periodontitis; SD, standard deviation; IV, inverse variance; CI, confidence intervals.

**Appendix 5**. **Pooled risk estimates between oral bacteria and periodontitis**

| **Sample size**  (Cases/controls) | **Bacterial species**  (>10^4^CFU/ml was defined as high level) | **Observed effect**  (OR, 95% CI/Mean±SD, mean bacterial level in case group vs control group) | **P values** |
| --- | --- | --- | --- |
| N=206 (136/70)  N=94 (68/26) | S. m  P. g  A. a | 0.82 (0.44-1.51)  3.38 (1.46-7.83)  1.36 (0.59-3.10) | 0.520  0.005  0.470 |
| N=112 (68/44) | L. B | 0.91 (0.32-2.60) | 0.860 |
| N=40 (27/13) | S. m  P. g | 1.02±1*10^4^ vs 3.16±3.46*10^5^  1.52±2.16*10^5^ vs 9.97±18.5*10^3^ | <0.05  <0.05 |

Note: N, number of subjects; CFU: Colony-Forming Units; OR: odds ratio; SD, standard deviation; CI, confidence intervals.

**Appendix 6**. **Pooled risk estimates between oral bacteria and periodontitis treatment**

| **Stages of treatment** | **Sample size**  (Post-/pre-treatment) | **Bacterial species**  (>10^4^CFU/ml was defined as high level) | **Observed effect**  (OR, 95% CI/Mean±SD, mean bacterial level in intervention group vs control group) | **P values** |
| --- | --- | --- | --- | --- |
| **Scaling and root planning** | N=92 (41/51) | S. m  P. g  F. n  A. a | 1.54 (0.47-5.02)  0.44 (0.17-1.10)  0.18 (0.02-1.72)  1.07 (0.42-2.73) | 0.470  0.080  0.140  0.880 |
|  | N=10 | S. m  P. g | 2.85±7.61*10^6^ vs 2.14±2.86*10^4^  6.64±8.23*10^3^ vs 1.27±3.40*10^6^ | 0.005  0.005 |
| **Periodontal surgery** | N=62 (14/48) | S. m  P. g  F. n  A. a | 0.87 (0.08-9.05)  0.73 (0.18-3.06)  0.87 (0.08-9.05)  0.45 (0.09-2.28) | 0.900  0.670  0.900  0.330 |

Note: N, number of subjects; CFU: Colony-Forming Units; OR: odds ratio; SD, standard deviation; CI, confidence intervals.

**Appendix 7.** **Funnel plot for caries risk in patients with periodontitis**

**
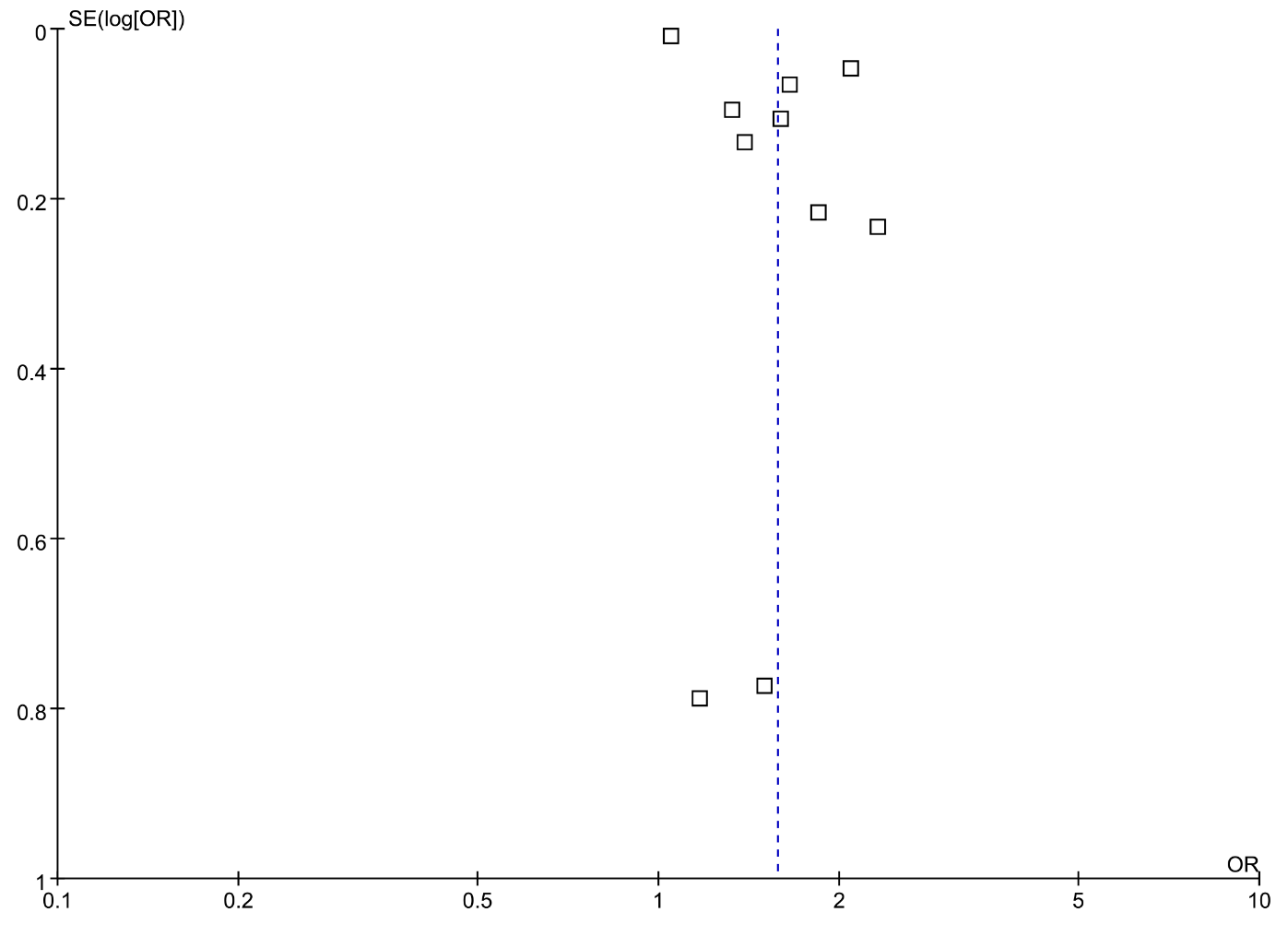
**

Note: SE, standard error; OR, odds ratio.
